# The effect of a hand hygiene intervention on the behaviour, practices and health of parents of preschool children in South Africa

**DOI:** 10.1177/17579139221123404

**Published:** 2022-09-21

**Authors:** Samantha Lange, Tobias George Barnard, Nisha Naicker

**Affiliations:** Water and Health Research Centre, Faculty of Health Sciences, University of Johannesburg, South Africa; Water and Health Research Centre, Faculty of Health Sciences, University of Johannesburg, Johannesburg, South Africa; Environmental Health Department, Faculty of Health Sciences, University of Johannesburg, Johannesburg, South Africa

**Keywords:** hand hygiene, parents, behaviour, intervention, diarrhoea, preschool, COVID-19

## Abstract

**Introduction:**

Diarrhoea and upper respiratory diseases are a leading cause of child mortality in children under 5 years of age both in South Africa and worldwide. Hand hygiene (HH) interventions play a critical role in reducing HH-related diseases, and the inclusion of all stakeholders in such interventions has improved the success of such interventions. The purpose of this study is to determine the effect of an HH intervention on the behaviour, practices, and health of parents of preschool children.

**Methodology:**

Seventeen preschools were randomly selected and placed into intervention (IG = 8) and control groups (CG = 9). Parents (*N* = 191) were requested to complete questionnaires both pre- and postintervention. An intervention was applied to IG preschool respondents. The data were analysed and compared pre- and postintervention between IG and CG.

**Results:**

Parents of IG showed a significant difference pre- and postintervention in HH practices such as washing hands after coughing and sneezing, and after using the toilet while parents in CG also indicated significant differences in HH practices of washing hands after coughing and sneezing, and after wiping children’s noses. Postintervention, IG families reporting runny tummies were significantly less than pre-intervention and a decrease in doctor’s visits. There was a 5% improvement of all HH practices in both IG and CG.

**Conclusion:**

Over 90% of parents in both groups washed hands after using the toilet, both pre- and postintervention. All HH practices for both groups showed increases both pre- and postintervention. By making use of available resources and regular communication with parents of preschoolers they are able to make the small changes necessary to improve their HH and that of their families.

**Graphical abstract:**
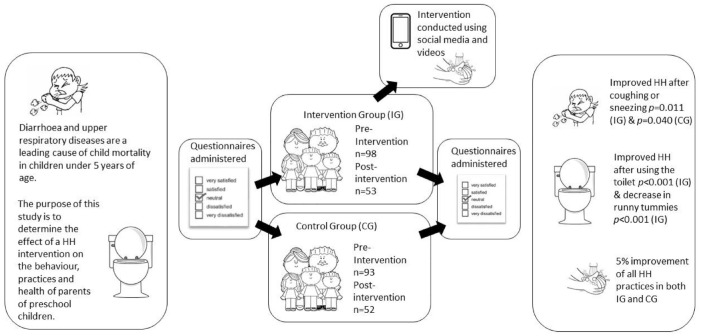
This is a graphical representation of the abstract.

## Introduction

It is well-documented that diarrhoea and upper respiratory infections are leading causes of death in children under the age of 5 years both in South Africa and worldwide.^1–4^ Both of these diseases have been linked to hand hygiene (HH) and studies have shown that such HH-related diseases can decrease as a result of improved HH practices.^5,6^ Improved HH forms part of water, sanitation and hygiene (WASH) initiatives most commonly implemented in communities and are implemented singularly or together in an attempt to reduce HH-related diseases and improve the health and wellbeing of communities.^7^ In the case of water and sanitation, interventions most often include the provision of infrastructure, the improvement of infrastructure or the implementation of methodologies to provide safe water. Hygiene interventions are broader and can take the form of provision of HH materials such as soap and hand sanitizer; education of communities including parents, school children and educators; and practical demonstrations and distribution of information.

Research has shown that the success of interventions is determined through the inclusion of the people who are affected by poor HH.^8^ In a preschool setting, caregivers at the school spend the majority of the day with the children, with parents taking over the responsibility of caring for the child once the child is at home after school or over weekends and school holidays. Therefore, the persons who would play a role in not only improving the HH of the child but also in providing an enabling environment for the child are the caregivers at the preschool and the parents. Many parents seem unaware of the vulnerability of children’s health, that HH could reduce disease transmission and not washing their hands after handling their children’s faecal waste or before feeding them.^9,10^ In an observational study of infants under 2 years, conducted in rural Zimbabwe, researchers reported that 30.0% of the primary caregivers (mostly mothers) of these children had visibly dirty hands, washed their hands 44.0% of the time but only used soap in 6% of those times, with 50.0% of the caregivers’ hands being contaminated with *Escherichia coli*.^11^

While reviewing literature on HH interventions, the interventions which proved successful where those that included not only the child but also the caregiver or parent or both.^7,12,13^ This article examines the effect that a simple intervention, which was conducted on preschool children, their school caregivers and their parents, would have on the parent’s practices, behaviour and health.

## Methodology

### Study population and sample selection

Seventeen preschools who were randomly selected and who agreed to participate in the study formed the sample population. The preschools were all in the Kempton Park area, which is a primarily residential part of Ekurhuleni, a metropolitan municipality in Gauteng, South Africa. The schools were randomly selected from a list compiled by the local environmental health division and comprised of schools which complied with the legislative prescripts of national and local legislation. Each school was approached, and the study was outlined to the principal of the school. When the principal agreed to participate, information packs were left at the schools to be distributed to the selected sample class. The preschools normally had one class for preschool children aged 4–5 years, although in two schools there were two classes, and all these classes were included in the study.

### Data collection

Data for the study was collected between February and November 2019, prior to COVID-19 being declared a pandemic by the World Health Organization (WHO) on 11 March 2020. The 17 participating preschools were randomly placed into an intervention group (IG) or a control group (CG) and were blinded to which group they belonged. Parents received an information pack with consent forms, an assent form for the child to participate and a household questionnaire for the parent to complete. This study included children aged 4–5 years; therefore, care was taken to develop information and assent letter that children could understand making use of pictures. Consent was given by the parents to allow the child to participate in the study and the child gave assent to the participation by placing a sticker on the form.

Questionnaires were returned to the school by the parent and collected by the researcher. The household questionnaire completed by the parents had three sections. The first section requested demographic information such as age, gender, educational status, age of children and number of persons in the household. There was also a request for structural information relating to the number of wash hand basins (WHBs) and toilets per household. The second part of the questionnaire dealt with the HH practices of the parents and their perceptions of their children’s HH practices. The statements were required to be answered in a 7-point Likert-type scale, with ‘0’ as never and ‘6’ as always. The final section dealt with the HH-related health of the respondent and their family. Respondents were required to answer yes or no to questions such as ‘Have you had a runny tummy in the past month?’ of ‘Have members of your family been to a doctor for any of these symptoms in the past month?’. The same household questionnaire, which had been completed by all parents in both groups prior to the intervention, was then administered to them approximately a month after the intervention was completed.

### HH intervention

**Figure 1:**
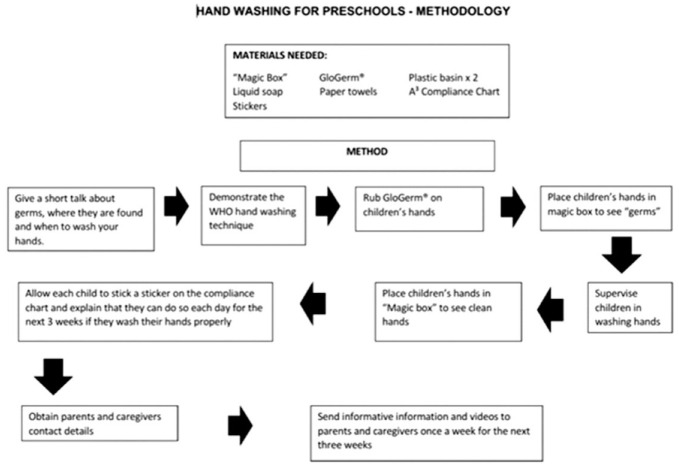
Flow diagram of methodology for HH intervention.

The information requested from the parents included a request for email addresses and cellular telephone numbers so that the parents could be contacted by the researcher. In the case of the intervention group parents received a number of health messages sent electronically to them for 3 weeks after the intervention as described in Figure 1 was conducted at the preschools with their children. They were also sent information in the form of a video showing them how the intervention was conducted at the preschools with their children as well as a WHO video demonstrating the correct way to wash hands.

### Analysis of data

The results were captured on an excel spreadsheet and then exported to SPSS where they were checked again for accuracy before analysis. Results were analysed for frequencies and answers were displayed in percentages. The answers for ‘almost always’ and ‘always’ were added together to provide a combined percentage. Comparisons were made in IG and CG pre- and postintervention as well as between IG and CG parents postintervention. Several of the statements in the questionnaire are attributed to activities which could help prevent the spread of colds and flu and diarrhoea. These statements have been linked together to form two new variables. These variables were tested for reliability using Cronbach’s Alpha and labelled: ‘Activities to prevent the spread of diarrhoea’ (α = 0.702) and ‘Activities to help prevent the spread of colds and flu’ (α = 0.572). Only parents who completed the questionnaire and the statements pre- and postintervention (*N* = 102) were included for this specific analysis. All results were subjected to an independent *t*-test and significance of results was determined as *p* = 0.05.

## Results

Pre-intervention there were 191 (52.5%) parents at the selected preschools who completed the questionnaire and consented to be part of the study. The IG consisted of 98 (51.0%) parents and 93 (49.0%) parents in CG. Parents completing the questionnaire postintervention were 105 (55.0%) with 53 (54.0%) IG parents and 52 (56.0%) CG parents completing the questionnaire postintervention. The demographic information was reported according to gender, age and education level of the respondent and is reported in Table 1. The majority of respondents were female in both IG (77.4%–78.0%) and CG (76.0%–80.8%) and mostly in the 30–49 years’ age group. Close to 80% of respondents in both groups have a tertiary education. There was a significant difference between the number of household members in IG and CG (*p* = 0.023); however, there was no other differences found between the groups in any of the other socio-demographic categories. On average there was one 4- to 6-year-old child although some households indicated two to three children in this age group.

**Table 1:** Demographics of respondents of IG and CG.

	IG, *n* = 98 (51%)	CG, *n* = 93 (49%)	*p* value*
Gender			0.949
Male	18.0%	18.0%	
Female	78.0%	76.0%	
Age			0.495
18-29 years	22.8%	18.8%	
30-49 years	76.1%	81.2%	
50-65 years	1.1%	0.0%	
Level of education			0.333
Primary	0.0%	0.6%	
Secondary	16.7%	17.3%	
Tertiary	82.2%	79.2%	
Other	1.1%	2.9%	
Number of household members			0.023
Mean	4.3	4.7	
Minimum	2	2	
Maximum	14	6	
Number of children < 6 years in household			
Mean (0–18 months)	1.1	1.0	0.419
Minimum (0–18 months)	1	1	
Maximum (0–18 months)	2	1	
Mean (19 months–3 years)	1	1.1	0.160
Minimum (19 months–3 years)	1	1	
Maximum (19 months–3 years)	1	2	
Mean (4–6 years)	1.1	1.1	0.968
Minimum (4–6 years)	1	1	
Maximum (4–6 years)	2	2	

CG: control group; IG: intervention group.

*p<.05.

Respondents were asked to indicate the number of toilets and WHBs available for the household. There was no significant difference between the IG and CG regarding HH facilities available in the households with a minimum of one toilet and one WHB available for each household. The maximum number of toilets per household could be found in CG where some households had six toilets. In IG, there were households with eight WHB although the mean for both groups was 2.2.

## Parents HH Practices

Table 2 indicates the responses of parents pre- and postintervention for IG and CG. An overall improvement in HH practices of 5.7% in IG and 6.88% in CG was reported.

**Table 2:** HH practices and HH related symptoms of parents pre- amd post-intervention

	IG Pre, *n* = 98 (51%)	IG Post, *n* = 53 (54%)	*p* value^a^	CG Pre, *n* = 93 (49%)	CG Post, *n* = 52 (56%)	*p* value^b^	IG & CG Post *p* value^c^
HH practices							
My children have become sick because I did not wash my hands correctly	3.3%	0.0%	0.001	4.9%	1.9%	0.001	0.555
I wash my hands correctly even when I am very busy	53.2%	55.8%	0.054	48.8%	51%	0.099	0.459
I wash my hands after coughing or sneezing	19.2%	35.8%	0.011	22.4%	22.6%	0.040	0.471
I wash my hands after wiping my children’s noses	31.9%	53.8%	0.081	28.9%	43.4%	0.043	0.881
I throw a tissue away once I have used it and do not keep it in my hand	62.8%	69.2%	0.179	63.8%	80.7%	0.182	0.329
I wash my hands after I have been to the toilet	94.7%	96.2%	0.000	90.3%	90.5%	0.008	0.637
I wash my hands after treating a cut or wound	86.1%	90.4%	0.000	84.3%	92.5%	0.003	0.530
I wash my hands before treating a cut or wound	69.1%	61.6%	0.066	63.4%	65.4%	0.011	0.689
I wash my hands before giving my children medication	39.4%	46.2%	0.005	19%	47.2%	0.034	0.243
I wash my hands after giving my children medication	24.5%	33.7%	0.128	20.4%	30.2%	0.032	0.116
I wash my hands before preparing food	91.5%	98.0%	0.000	91.5%	90.6%	0.000	0.403
I wash my hands after touching household pets	37.3%	36.0%	0.000	43.3%	44.3%	0.000	0.676
HH practices (children)							
My children wash their hands after using the bathroom	64.6%	63.5%	0.019	59.3%	73.6%	0.008	0.254
My children wash their hands before eating	56.4%	75.0%	0.120	62.7%	61.6%	0.016	0.455
HH equipment							
I have the correct equipment to be able wash my hands correctly	80.3%	81.2%	0.000	67.1%	77.3%	0.621	0.502
There is soap and towels available at all basins for hand washing	85.4%	88.5%	0.008	81.4%	83.0%	0.000	0.849
I use soap when I wash my hands	75.6%	82.7%	0.001	73.5%	82.7%	0.001	0.573
I use a clean cloth to dry my hands	78.8%	78.9%	0.000	68.6%	75.0%	0.007	0.470
HH symptoms							
In the past month, I have experienced a runny tummy	25.0%	24.5%	0.305	29.8%	17.3%	0.013	0.363
In the past month, members of my family have experienced a runny tummy	42.1%	38.5%	0.000	37.3%	19.2%	0.429	0.013
In the past month, I have experienced cold symptoms such as coughing, sneezing, blocked nose	62.1%	56.6%	0.330	71.4%	55.8%	0.301	0.931
In the past month, members of my family have experienced cold symptoms such as coughing, sneezing, blocked nose	77.1%	66.0%	0.081	82.1%	82.7%	0.916	0.051
In the past month, I have experienced vomiting and a runny tummy	8.4%	13.2%	0.482	9.6%	7.7%	0.703	0.356
In the past month, members of my family have experienced vomiting and a runny tummy	21.1%	17.0%	0.931	24.1%	13.5%	0.292	0.616
In the past month, I have been to the doctor for treatment of any of the above symptoms	22.1%	17.0%	0.060	20.2%	25.0%	0.088	0.313
In the past month, members of my family have been to the doctor for treatment of any of the above symptoms	43.8%	20.8%	0.067	1.9%	34.0%	0.424	0.131

CG: control group; HH: hand hygiene; IG: intervention group.

a Difference IG pre- and postintervention.

b Difference CG pre- and postintervention.

c Difference IG & CG postintervention.

Parents of IG showed a significant difference pre- and postintervention in HH practices of washing hands after coughing and sneezing (*p* = 0.011); after using the toilet (*p* < 0.001); after treating a wound (*p* < 0.001); before giving medication (*p* = 0.005); before preparing food (*p* < 0.001), and after touching household pets (*p* < 0.001). There was a significant difference (*p* = 0.019) in IG postintervention where there was a 1.1% decrease in children washing hands after they had used the toilet.

Data for parents in CG also indicated significant differences in HH practices of washing hands coughing and sneezing (*p* = 0.040); after wiping children’s noses(*p* = 0.043); before eating(*p* = 0.001); after using the toilet (*p* = 0.008); before (*p* = 0.011) and after treating a wound (*p* = 0.003); before (*p* = 0.034) and after giving medication (*p* = 0.034); before preparing food (*p* < 0.001) and after touching household pets (*p* < 0.001). A difference (*p* = 0.016) was seen in CG with a 1.1% decrease of children washing hands before eating.

There was a significant difference between IG and CG respondents’ families reporting a runny tummy postintervention (*p* = 0.013) with CG reporting less than IG. However, postintervention, IG families reporting runny tummies were significantly less than pre-intervention (*p* < 0.001).

There was an average decrease in symptoms and treatments pre- and postintervention in IG of 6.0% and CG 7.3%. In a few instances respondents listed the symptoms that they had sought a doctor’s assistance for which were listed as cold and flu, diarrhoea and other illness. There was no significant difference pre and post between IG and CG regarding the listing of these symptoms. There was an increase in reported visits by respondents to doctors for colds and flu in CG postintervention but it was not significant (*p* = 0.983).

The overall average improvement in HH practices postintervention in parents of IG was 5.7% with a 6.8% improvement in CG.

## Activities to Help Prevent the Spread of Diarrhoea, Colds and Flu

There were 53 (51.9%) IG and 49 (48.0%) in CG respondents who were eligible to be part of the combined variables described in the methodology section. The combined variable of ‘Activities to prevent the spread of diarrhoea’ showed a significant difference (*p* = 0.035) in IG pre- and postintervention. In the combined variable dealing with ‘Activities to help prevent the spread of colds and flu’ CG showed a significant difference (*p* = 0.003) between pre- and postactivities.

A multivariate analysis for risk factors for the presence of diarrhoeal symptoms, including runny tummies and vomiting, and respiratory symptoms was performed. A stepwise process was followed. Significant associations for experiences of diarrhoeal symptoms were found for the educational status of the parent and number of household members. A higher level of education (tertiary and above) was significantly associated with a lower odds ratio (OR) of diarrhoeal symptoms (OR: 0.755; 95% confidence interval (CI): 0.009, 0.588; *p* = 0.014). Having more than four household members was significantly associated with a higher OR (OR: 1.187; 95% CI: 0.179, 0.980; *p* = 0.045). The same analysis was conducted in the group for respiratory symptoms such as coughing, sneezing and runny noses. No significant associations were found for these symptoms.

## Parents Comments in the Questionnaire

A comments section was provided for at the end of the questionnaires both pre- and postintervention for each group. In IG, there were 37 comments of which 13 (35.1%) were pre- and 24 (64.9%) were postintervention comments. Postintervention for IG 11 (46.0%) of the comments was related to the intervention and how well their children interacted with the intervention. These comments from parents included statements such as:

*Ooh, he was teaching me to wash my hands exactly the way you are doing it*

*I got a lecture as well about hand hygiene and germs and how to wash my hands from my daughter.*

In CG, there were 22 comments with 7 (31.8%) being pre- and 15 (68.2%) postintervention comments. There were 9 (40.9%) of comments in CG which were questionnaire related, that is, the participant had learned something from the questionnaire and the same amount (40.9%) which were HH-related comments. The remaining 18.2% of comments in CG were general comments, for example, ‘Good luck’.

As part of the questionnaire for IG postintervention parents were asked if they had learned anything new about HH and if they found the information useful. There were 52 (98.1%) of the 53 postintervention IG respondents who affirmed that they had learned something new about HH. Of the 53 IG respondents 35 (66.0%) answered the question as to whether they found the information useful or not with 9 (25.7%) answering ‘yes’; 24 (68.6%) answering ‘maybe’ and 2 (5.7%) parents answering ‘no’. This group of parents indicated that 46 (86.8%) of them were happy to receive HH health messages and information electronically.

## Discussion

Parents of IG and CG showed at least a 5% improvement in HH practices postintervention with CG improvement slightly higher (6.8%) than IG (5.7%). The improvements in IG as a result of the intervention correspond with similar research where parents have improved their own and their children’s HH.^14,15^ There could have been a form of social desirability bias in the answering of the questions, in that parents may have wanted to be seen by the researcher as a “good’ parent and therefore gave answers to the statements depending on what they thought the researcher would want to know. This type of social desirability was found among preschool children who displayed better HH when someone was accompanying them as a social influence.^8^ The notion of disgust, and being seen as a “dirty” person has also been reported as a driver for improved HH^16^ and similarly could have influenced the answers of the parents. If this is the case, it would indicate a good knowledge of HH practices initially and improved knowledge postintervention, based on, in the case of IG, the intervention and in the case of CG the possible prompting brought on by statements in the questionnaire which created a thought process for improvement in practices.^17^ There was a significant improvement in the nose-hygiene practices of parents in CG and a less-significant improvement in IG; however, there was also a decrease in respiratory illnesses in the families of both groups according to the reporting of the parents. Diarrhoeal disease–related practices improved significantly in IG and also improved in CG which was indicated in diarrhoeal illnesses and symptoms. Households with respondents with a tertiary education were 0.75 less likely to experience diarrhoeal symptoms. A similar study of primary school pupils in Wuhan showed that children of mother’s with a tertiary education were 0.68 times more likely to adhere to good HH, which was found to be significant.^18^ It is possible to draw an association between good HH and the parental level of education, with 82.2% of parents in IG and 79.2% in CG reporting a tertiary education.

Whether from social desirability or improved knowledge of HH and the effect it could have on the health of their children and families, parents of IG and CG both improved on their statement that their children could have become sick because they did not wash their hands properly (3%). This statement is confirmed by the significant improvements of CG in the respiratory infection-related activities (*p* = 0.003) and IG improvements in diahrroea-related activities (*p* = 0.035). There was a 23% and 7.9% decrease in doctor’s visits for IG and CG, respectively.

Parents of the IG were targeted with electronic messaging information as part of the intervention. A video showing how the children were taught to wash their hands was sent to the parents via WhatsApp. Almost all the parents (98.1%) indicated that they had learned something new from these messages. This feedback from parents indicated they were happy to receive health messages through electronic and social media (email and WhatsApp) and showed to be an effective way of increasing knowledge of parents and caregivers, as reported in previous studies.^10,19^

Information regarding use of hand sanitizers, critical times to wash hands, and how to wash hands was forwarded to the parents regularly during a 3-week period after the intervention. Comparing the pre- and postintervention data on HH, HH-related diseases, and doctor’s visits in IG could be influenced by the information received by the parents and also the influence of the children on their parents. This was reported as effective in an HH intervention where one of the sources of information for the parent was the passing of information from the school-going child, who has also been exposed to an intervention, to the parent.^20,21^ There have also been slight improvements found in the health of children under 5 with siblings attending schools who are part of an intervention.^7^

During analysis, many of the results showed significant improvements by CG and therefore, as the CG was not exposed to the intervention an explanation for the improvements in CG needs to be sought. Several possible explanations for these increases have been explored. Research of itself is a type of intervention, as from the time that a participant provides informed consent, whether they are in IG or CG, they are no longer in a usual situation as they are now research participants.^22^

Another explanation could be the testing effect which occurs when participants improve their scores in the postintervention test as they have learned from the statements or questions asked^22^ or the statements or questions have stimulated new thinking thereby acting as a type of intervention in IG and CG.^23^ This can be seen as a type of learning tool, which proved effective with students who were exposed to a pretest and who then performed better in a post-test than their counterparts who did not conduct a pretest.^24^ Some of the statements that formed part of the questionnaire for parents could be a driver for behaviour change or provide an opportunity to stimulate thinking with regard to hygiene, which could explain the improvements in both IG and CG.

A third effect which could affect the improvement of results in both IG and CG is a reactivity of measurement whereby mere fact of being measured against a previous measurement may provide an improvement.^22^ The questionnaires were administered approximately 4 months apart and contained the same statements pre- and postintervention, providing a possibility for participants to recall and improve on their previous score.

The importance of continued HH interventions has been highlighted during the COVID-19 pandemic. Keeping in mind that pre-COVID-19, correct hand washing was practised by 19% of the world’s population^6^ one would expect vast improvements as a result of the emphasis placed on hand washing as a means of preventing transmission of COVID-19. Numerous studies have shown that there was a significant decrease in HH compliance over time, once the initial crisis period had passed, despite the pandemic. These studies indicated that healthcare workers reverted back to old HH habits and practices, sometimes within 14–20 weeks of initial restrictions and school closures implemented as preventive measures for the pandemic.^25,26^ These studies show that even in the face of a pandemic where hand washing is indicated as a primary preventive tool, continued reinforcement through effective interventions are needed to improve and maintain HH compliance in all sectors.

## Strengths and Limitations

The study provided similar results to previous studies which indicate that interventions can improve HH and reduce HH-related diseases.^27,28^ The study provided numerous significant results while following the published study protocol,^29^ validating the protocol.

As with any HH intervention study the major weakness of the study was the lack of blinding of study participants.^30^ There was partial blinding in that participants were not aware of all aspects of the study or its anticipated outcomes. Participants were also not aware of an IG or CG nor did they know which group they were part of.

A limitation of the study was that due to time and manpower constraints, the study did not start simultaneously in all preschools but was a rolling start in that some schools enrolled earlier than others. The study used diarrhoea and upper respiratory tract infections as an indicator, but data were collected based on parents own perceptions of these to symptoms.

## Conclusion

HH practices of these parents were considered to be of a high standard compared to the previously stated practice of 19% of persons worldwide washing hands with soap after defecating.^31^ Over 90% of parents in both groups washed hands after using the toilet, both pre- and postintervention. All HH practices for both groups showed increases both pre- and postintervention. The knowledge of the parents increased as was shown by how they answered the question as to whether their children had become ill due to their own hygiene practices. Pre-intervention parents answered this statement by denying that their actions had health implications for the children; however, postintervention parents in IG and CG answered the statement in a way that implied that they had come to the realisation that they may have contributed to children’s illnesses through their HH practices. Simple interventions such as the one administered to the respondents in this study is a cost-effective way of educating parents and improving HH in the home as well as improving the health of young children. Administering questionnaires can be a form of intervention and stimulation of knowledge and learning. By making use of available resources such as simple WhatsApp messages and regular communication with parents of preschoolers, they are able to make the small changes necessary to improve their HH and those of their families.

## Recommendations for Further Research

A recommendation regarding further study would be to determine the HH practices of preschool parents as a result of the pandemic, as it has been previously mentioned that HH compliance improves during epidemics, or in this case, a pandemic.^32^
